# Prevalence of age-related macular degeneration in Iran and its projections through 2050: a systematic review and meta-analysis

**DOI:** 10.1186/s12886-023-03218-3

**Published:** 2023-11-25

**Authors:** Amirhossein Roshanshad, Romina Roshanshad, Seyed Ali Moosavi, Ali Ardekani, Sara Sadat Nabavizadeh, Reza Fereidooni, Hossein Ashraf, Hossein Molavi Vardanjani

**Affiliations:** 1 Poostchi Ophthalmology Research Center, Shiraz University of Medical Sciences, Shiraz, Iran; 2 MPH Department, Shiraz University of Medical Sciences, Shiraz, Iran; 3 Student Research Committee, Shiraz University of Medical Sciences, Shiraz, Iran; 4 School of Medicine, Shiraz University of Medical Sciences, Shiraz, Iran; 5 Otolaryngology Research Center, Shiraz University of Medical Sciences, Shiraz, Iran; 6 Health Policy Research Center, Institute of Health, Shiraz University of Medical Sciences, Shiraz, Iran; 7 Research Center for Traditional Medicine and History of Medicine, Shiraz University of Medical Sciences, Shiraz, Iran

**Keywords:** AMD, Epidemiology, Iran, Macular Degeneration, Middle East, Prevalence

## Abstract

**Background:**

Age-related Macular Degeneration (AMD) is one of the most common causes of vision loss. A substantial increase in the burden of AMD is expected in the aging populations, including the Iranians. We investigated the age and gender-specific prevalence of AMD and its determinants in Iran.

**Methods:**

We systematically searched international (PubMed, Scopus, Embase, etc.) and local (IranDoc, Magiran, etc.) online databases. We included cross-sectional or cohort studies, either clinic- or population-based, published on the prevalence of AMD among Iranians, with no limitation on age. Joanna Briggs Institute (JBI) tools for critical appraisal were used. Prevalence estimates are pooled by applying random-effects modeling. Subgroup analysis and meta-regression were performed.

**Results:**

Seventeen studies with 16,120 participants were included. Based on studies in general population, the pooled prevalence of AMD was 10.8% (95% CI: 6.5-16.2%) in males, and 9.8% (95% CI: 4.7-16.4%) in females. 8.5% of moderate vision impaired, 13.6% of severe vision impaired, and 15.7% of blind participants were affected by AMD. The prevalence of AMD was 2% in 40–49, and 32.3% in the ≥ 80 population. The prevalence of AMD was 11.9% among the visually impaired vs. 8.7% in the general population. The study’s sampling method, location, and mean age were correlated with the heterogeneities of the prevalence. We observed an increasing trend in the number of AMD cases (average annual percent change = 3.66%; 95% CI: 3.65–3.67%) from 1990 to 2050. The expected number of AMD cases in Iran will be near 5.5 million by 2050.

**Conclusion:**

The prevalence of AMD in Iran was somewhere between the prevalence of Asians and Europeans. Given the aging trend of the Iranian community and an average annual percent change of 3.66%, it is indispensable to adopt preventive and screening policies to diminish the burden of the disease in the future decades.

**Supplementary Information:**

The online version contains supplementary material available at 10.1186/s12886-023-03218-3.

## Introduction

Age-related macular degeneration (AMD) is a progressive retinal disease that involves the macula and might lead to irreversible visual impairment [1, 2]. The vision impairment and blindness caused by AMD are avoidable if diagnosed early [3, 4]. The development of age-related macular degeneration (AMD) may be influenced by various demographic and environmental factors, including aging, cigarette smoking, previous cataract surgery, and family history of AMD [5]. Consideration of these risk factors could help to better understand the variations in disease prevalence across different countries. In addition to its notable prevalence, effectively managing AMD requires significant time and resources, leading to increased demand for services and financial reimbursements [6].

AMD was the third most common cause of moderate to severe vision loss in 2015, affecting 8.4 million visually impaired individuals, globally [7]. It is estimated that 5.6% of all causes of blindness in the world can be attributed to AMD [3]. A meta-analysis conducted by Li et al. predicted that in 2050, more than 77 million people will be influenced by any type of AMD in Europe [8]. In a study published in 2020 in China, it is projected that macular degeneration accounts for 3·4% of all causes of moderate vision impairment in 1990; however, this proportion increased to 4·6% in 2019 [8]. In North Africa and the Middle East (NAME) region, AMD is responsible for 8.3% of all causes of blindness [3], indicating the higher prevalence of AMD in the NAME region, compared to the global prevalence.

Iran, the most populated country in the Middle East [9], is facing an unprecedented increase in the aging population. According to previous studies, the proportion of people aged more than 60 has escalated in the past few decades from 7.3% to 2006 to 8.6% in 2016. It is projected that this proportion will reach 10.5% in 2025 and 21.7% in 2050 [10, 11]; consequently, a substantial increase in the prevalence and burden of age-related diseases, including AMD is predicted.

Currently, there is no systematic review and meta-analysis regarding the prevalence of AMD in Iran. Herein, we performed a meta-analysis of the prevalence of AMD in the Iranian population and its predictors to better approximate the disease burden in Iran.

## Methods

### Study protocol

We adhered to the Preferred Reporting Items for Systematic Reviews and Meta-Analyses (PRISMA) statement to conduct this study [12]. Our study protocol was registered in PROSPERO (ID: CRD42021244150).

### Eligibility criteria

The main goal of the current study is to find the epidemiologic characteristics of AMD in the Iranian population. We categorized the results of the study into four sections: (1) Prevalence of AMD in the Iranian population, (2) meta-analysis of the pooled prevalence of AMD, (3) subgroup analysis of the prevalence of AMD, (4) estimation of the population affected with AMD in Iran by 2050.

#### Inclusion criteria

The original articles including the observational studies with the following criteria were included in our meta-analysis:

- Studies in which the prevalence of patients with the outcomes of interest was presented or could be obtained, with no limitation on age.
- Studies with acceptable study design for a prevalence study, either a cross-sectional or a cohort study.
- Studies with adequate information on study design, characteristics of the sample, age group of the participants, study location, and the method used to diagnose AMD.
- Sample of the participants was drawn from the Iranian population, either clinic-based or population-based.

#### Exclusion criteria

- Review articles, case reports, case series, case-controls, and conference abstracts.
- Population size less than 20.

### Search strategy and study selection

We searched for publications that presented the prevalence of AMD among Iranians. We searched the databases of PubMed, Scopus, Embase, Web of Science, and Google Scholar with the following keywords: “Iran” AND “AMD OR Macular degeneration” AND “Prevalence OR epidemiologic”. The detailed search strategy is presented in Table S1 of the Supplementary file. We also searched local databases of Scientific Information Database (SID), IranDoc, and Magiran to find more relevant studies. The initial search was conducted in June 2021 and was updated in October 2022 and we included the studies published from inception to the search date. Both English and Persian keywords for macular degeneration were searched in local databases. Besides, we manually checked medRxiv and Research Square databases for preprints and gray literature. No restriction was considered to limit the search results. Reference lists of the included studies were also assessed to find more relevant publications. Furthermore, the publications of the authors were searched separately to ensure the generalizability of the search protocol. Two authors, A.A. and R.F., independently searched the literature and consensus was achieved by consultation with the third author, A.R.

### Study selection

First, we gathered identified studies from different databases and sources and removed the duplicates. Several studies were conducted using the same target population with similar study designs and sample sizes, yielding very close prevalence estimates. In such cases, studies with higher sample sizes and more detailed data were selected. Thereafter, titles and abstracts were screened to filter irrelevant studies. Finally, full texts were assessed based on the inclusion and exclusion criteria to distinguish eligible studies. Two authors, S.S.N. and S.A.M., independently screened the titles and abstracts and identified the eligible studies. The third author, A.R., was consulted in case of disagreements.

### Data extraction

The following data were extracted from each included study: the first author, year of publication, study design, sampling method, study location (province/city), sample size, mean age and age group of the participants, male to female (M/F) ratio, the severity of vision impairment and the vision assessment method (best corrected visual acuity [BCVA] or presenting visual acuity [PVA]). Studies with random, stratified, and systematic sampling were categorized as random sampling, while studies with convenience, quota, judgment, and snowball sampling were classified as non-probability sampling [13]. In Shirzadi et al. study, nearly 88% of the participants had vision impairment, which is much higher than the prevalence of visual impairment in Iran (5.57%) [14]. The sample of this study was much more representative of a visually impaired population than a general population. The human development index (HDI) of the study location was retrieved from the Subnational Human Development Index (4.0) [15]. HDI of the study location was categorized into 5 groups, very high, high, medium, low, and very low. As the HDI of some locations and cities were not provided on the mentioned website, we also consulted an experienced epidemiologist for better determination of the HDI categories of the cities. The number of AMD patients among males, females, visually impaired candidates, and all participants was extracted to calculate the prevalence of AMD. Finally, we used the data provided by United Nations Population Division (UNPD) to import the age-specific population of Iran from 1990 to 2020 in 5-year intervals for each 10-year age group (40–49, 50–59, 60–69, 70–79, > 80). Similarly, we obtained the estimated age-specific population of Iran for each of these 10-year age groups from 2020 to 2050. Data extraction of the included studies was accomplished by two independent reviewers (A.R. and R.R.) and disagreements were discussed with a third author (H.M.V.).

### Assessment of the risk of bias

The quality of the included articles was assessed by two independent investigators (A.R and R.R) with Joanna Briggs Institute (JBI) tools for assessment of the risk of bias in cross-sectional and cohort studies [16]. According to the scores, the studied were classified as low, medium, and high quality. In the case of discordances, consensus was achieved by consultation with a third author (H.M.V.). Each item of the tool was scored 1, 0, and − 1 for “Yes”, “Unclear”, and “No” responses, respectively. The sum of the scores was calculated for all the studies. Studies with scores of less than 0, 1 to 4, and more than 4 were considered to have a high, medium, and low risk of bias. The details of the risk of bias assessment are presented in Table S2 of the Supplementary file.

### Statistical analysis

Heterogeneity between studies was assessed using I^2^ statistics. I^2^ values of 25, 50, and 75% were selected to reflect low, medium, and high heterogeneity, respectively [17]. Random-effects meta-analysis was performed. We used a DerSimonian-Laird random effect model to perform the analysis. Accordingly, the pooled prevalence of participants with diagnosed AMD with its 95% confidence interval (CI) was calculated. Subgroup analysis was applied based on different variables, e.g. methods of sampling, HDI of the province, population-based or clinic-based study, male-to-female ratio, mean age of the participants, urban or rural population, and whether the sample was drawn from a normal population or vision impaired patients. Meta-regression was performed to find which variables can affect the prevalence of AMD. We calculated the age-specific and total number of AMD cases by multiplying the age-specific prevalence rates for each 10-year age group with the imported or estimated population of Iran for the corresponding 10-year group, obtained from UNPD [18]. To calculate the average annual percent change (AAPC), Poisson regression was used to calculate the incidence rate ratio of AMD cases from 1990 to 2050. Sensitivity analysis was performed to detect the individual effects of any single study. Furthermore, we performed another sensitivity analysis by including population-based studies and excluding clinic-based studies. Finally, we assessed the publication bias using funnel plots and Eager’s tests. Using Stata version 14.0 software (Stata Corporation, College Station, TX, USA). A p-value of < 0.05 was considered as statistically significant.

### Ethical considerations

After approval by the ethics committee of Shiraz University of Medical Sciences (ethical code: IR.SUMS.MED.REC.1400.597), this study was conducted with regard to the tenets of the Declaration of Helsinki.

## Results

### Inclusion of the studies

**Fig. 1:**
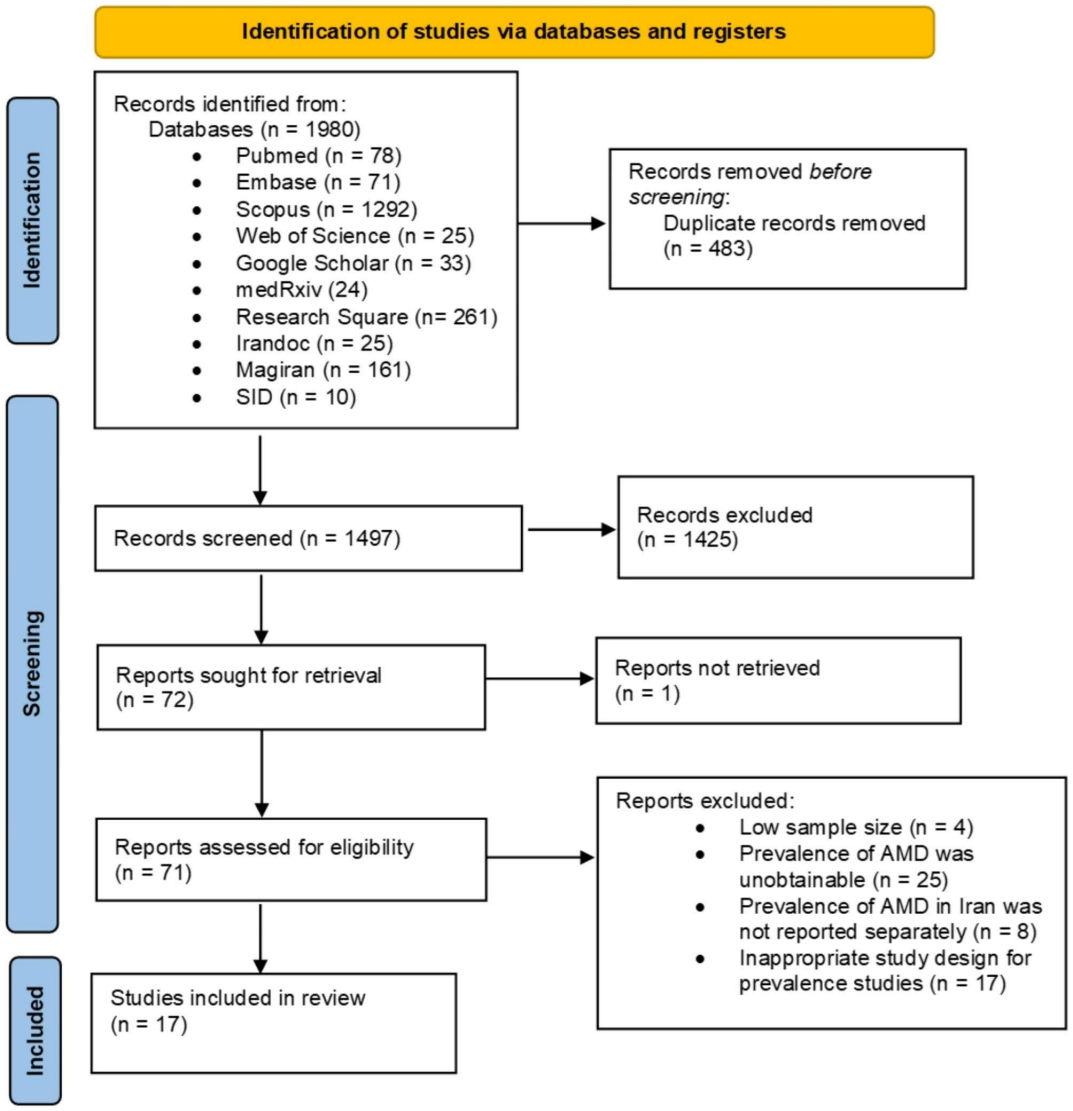
Flow diagram of the included studies evaluating the prevalence of AMD in the Iranian population

A total of 1466 articles were identified through a computerized search of PubMed, Embase, Web of Science, and Scopus. Searching the reference lists, Google Scholar, IranDoc, Magiran, and SID yield 514 more entities. After removing the duplicates, we screened titles and abstracts of 1497 unique studies. 1425 of these 1497 articles were irrelevant, remaining 72 studies for full-text evaluation. 55 of these 72 articles were excluded based on our selection criteria. Therefore, 17 articles with 16,120 participants were eligible to enter the study (Fig. 1) [19–35]. Of these 17 studies conducted in eight different provinces of Iran, 12 were population-based and five studies included clinic patients. Four of the studies used non-probability sampling methods and 12 of them used probability sampling methods. Eight of the studies reported the prevalence of AMD in females and males, separately. Details about the characteristics of the included studies are presented in Table 1. The details of the risk of bias assessment are presented in Table S2 of the Supplementary file.

**Table 1:** Characteristics of the included studies evaluating the prevalence of age-related macular degeneration in the Iranian population

First author	Year	Design	Sampling	Population or clinic	Province (City)	Age group	Number of participants	Visual status of the population	Definition of VI	Risk of bias
Fotouhi [22]	2004	C/S	Stratified cluster sampling	Population-based	Tehran (Tehran)	All age	75	Visually impaired population	BCVA	Low
Riazi [32]	2005	C/S	Non randomized sampling	Clinic patients	Tehran (Tehran)	All age	468	Visually impaired population	PVA	High
Hatef [26]	2008	C/S	Stratified random cluster sampling	Population-based	Tehran (Tehran)	5–96	4354	General population		Medium
Rajavi [30]	2011	C/S	Multistage cluster systematic random sampling	Population-based	Tehran (Varamin)	≥ 50 years	275	Visually impaired population	PVA	Low
Sharifi [33]	2013	C/S	Sequential sampling	Clinic patients	Kerman	20–78	1061	Visually impaired population		Medium
Yekta [35]	2013	C/S	Random cluster sampling	Population-based	Mazandaran (Sari)	≥ 55 years	31	Visually impaired population	BCVA	Medium
Akhgary [19]	2014	C/S	Non-probability sampling	Clinic patients	Tehran (Tehran)	7–90	204	Visually impaired population	BCVA	High
Nodehi [29]	2015	C/S	Non-probability sampling	Clinic patients	Tehran (Tehran)	≥ 60 years	392	General population		High
Hashemi [23]	2015	C/S	Multistage cluster sampling	Population-based	Semnan (Shahroud)	40–64	4387	General population		Medium
Katibeh [28]	2015	C/S	Cluster random sampling	Population-based	Yazd	40–80	108	Visually impaired population	BCVA	Low
Rasoulinejad [31]	2015	C/S	Census	Population-based	Mazandaran (Amirkola)	60–89	505	General population		Low
Hashemi [24]	2017	C/S	Randomized cluster sampling	Population-based	Mazandaran (Sari)	55–87	937	General population		Low
Katibeh [27]	2017	C/S	Multistage cluster sampling	Population-based	Gilan	≥ 50 years	344	Visually impaired population	PVA	Medium
Hashemi [25]	2018	C/S	Random stratified cluster sampling	Population-based	Khorasan (Mashhad)	1–90	62	Visually impaired population	BCVA	Low
Ashrafi [20]	2019	C/S	Multistage systematic cluster random sampling	Population-based	Kurdistan	50–99	414	Visually impaired population	PVA	Low
Behboudi [21]	2020	C/S	Cluster sampling	Population-based	Gilan	50–94	2275	General population		Low
Shirzadi [34]	2020	C/S	Random sampling	Clinic patients	Tehran (Tehran)	9–82	228	Visually impaired population	BCVA	Medium

BCVA: Best corrected visual acuity; PVA: Presenting visual acuity

### Prevalence of AMD in the Iranian population

**Fig. 2:**
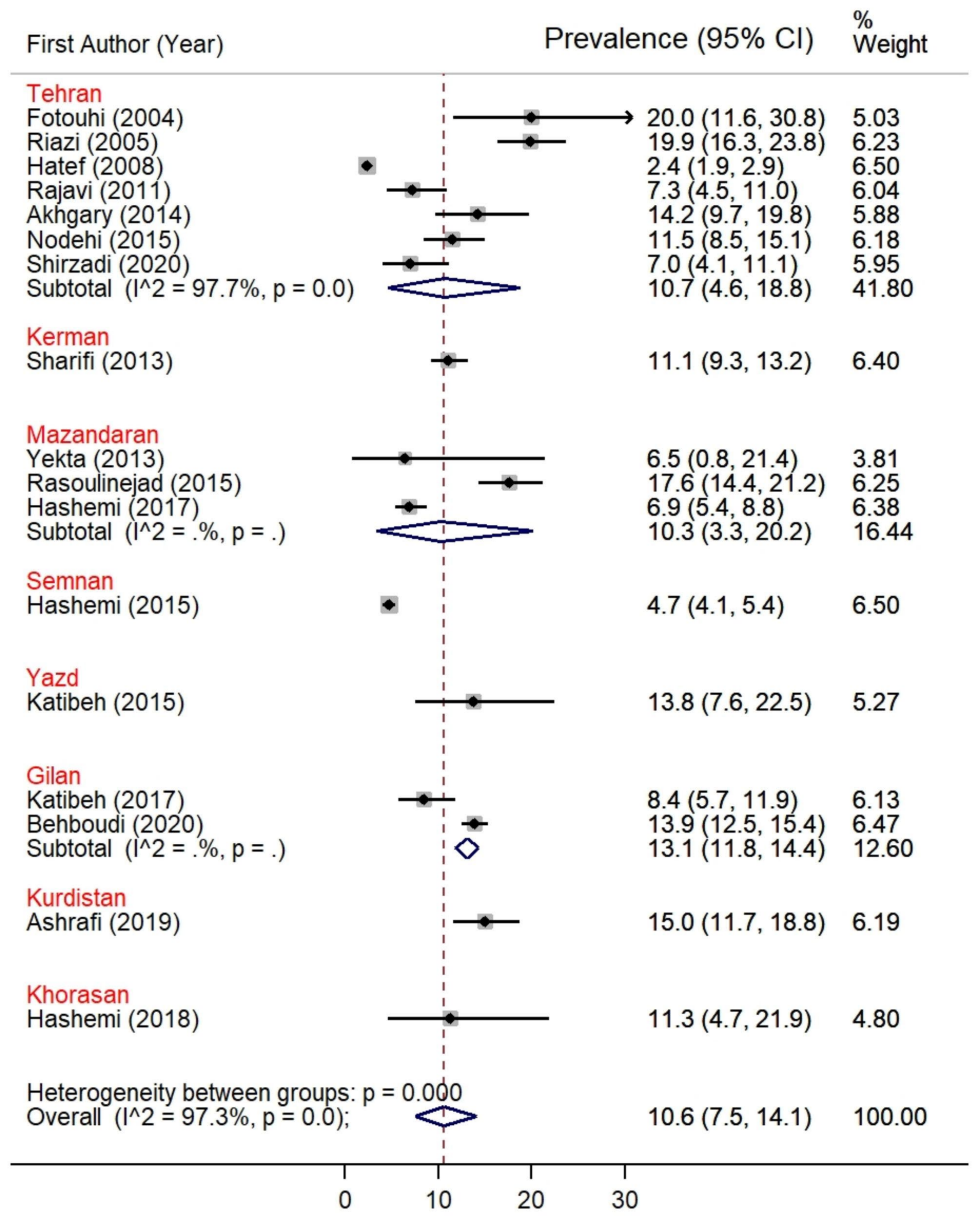
Forest plot diagram showing the prevalence of AMD in the Iranian population and the associated 95% CI in different provinces of Iran

The prevalence of AMD in the Iranian population ranged from 2.4 to 20.0%. Fotouhi et al. and Riazi et al. studies reported the highest prevalence of AMD among the included studies, 20.0% (95% CI [11.6-30.8%]) for Fotouhi et al. study and 19.9% (95% CI [16.3-23.8%]) for Riazi et al. study of the visually impaired participants were diagnosed with AMD [22, 32]. The lowest prevalence of AMD was observed in Hatef et al. study, which was 2.4% (95% CI [1.9-2.9%]) [26]. Interestingly, all these three studies were conducted in the capital of Iran, Tehran. (Fig. 2) Behboudi et al. study was the only study to report the overall prevalence of AMD (13.9%), early (13.3%), and late (0.6%) AMD altogether [21]. After standardizing for age and sex, the rates of any, early, and late AMD were 13.9%, 13.2%, and 0.7%, respectively. The reported prevalence of AMD was 10.7% in Tehran, 11.1% in Kerman, 10.3% in Mazandaran, 4.7% in Semnan, 13.8% in Yazd, 13.1% in Gilan, 15.0% in Kurdistan, and 11.3% in Khorasan province.

### Meta-analysis of the pooled prevalence of AMD

In the general population, the pooled prevalence of AMD was 10.8% (95% CI [6.5-16.2%]) in males and 9.8% (95% CI [4.7-16.4%]) in females with no significant sex difference (p-value = 0.06). Furthermore, 8.5% (95% CI [4.8-13.2%]) of moderate vision impaired, 13.6% (95% CI [7.9-20.3%]) of severe vision impaired, and 15.7% (95% CI [6.1-28.2%]) of blind participants were affected by AMD (Table 2). We also calculated the prevalence of AMD in different age groups, 0.0% in less than 40, 2.0% in 40–49, 5.7% in 50–59, 9.1% in 60–69, 19.6% in 70–79, and 32.3% in more than 80 years old population.

**Table 2:** Prevalence of age-related macular degeneration in the Iranian population in different age, gender, and vision status subgroups

Variables	Subgroups	Prevalence % (95% CI)
Age group	< 40	0 (0, 0)
	40–49	2 (1.3, 2.7)
	50–59	5.7 (2.4, 10.1)
	60–69	9.1 (5.2, 13.7)
	70–79	19.6 (11.3, 29.6)
	> 80	32.3 (13.5, 54.6)
Gender	Male	10.8 (6.5, 16.2)
	Female	9.8 (4.7, 16.4)
Severity of vision impairment	Moderate	8.5 (4.8, 13.2)
	Severe	13.6 (7.9, 20.3)
	Blind	15.7 (6.1, 28.2)

### Subgroup analysis of the prevalence of AMD

**Fig. 3:**
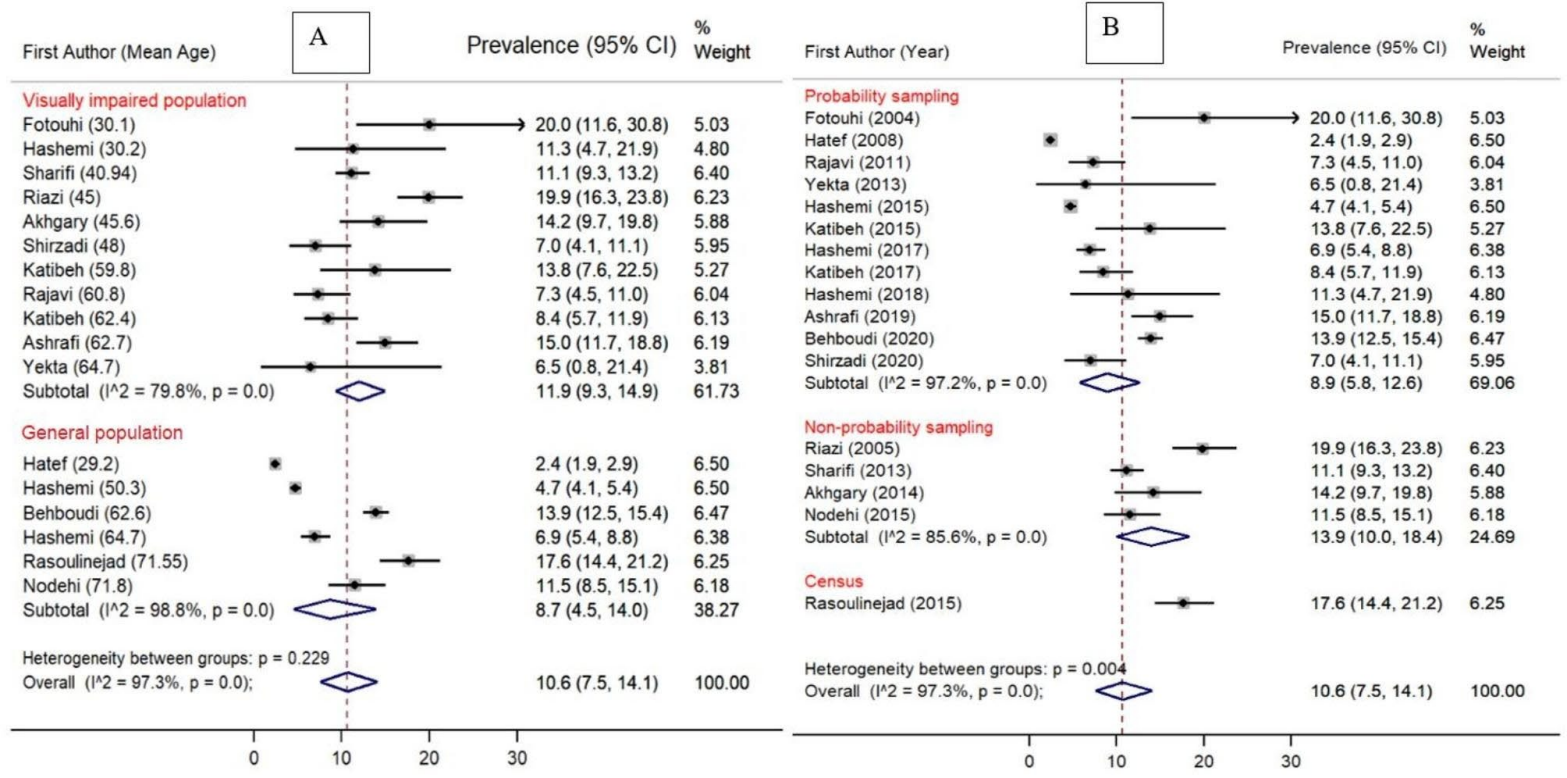
Forest plot diagram showing the subgroup analysis of the prevalence of AMD in the Iranian population and the associated 95% CI based on: (**A**) Vision status of the target population [Prevalence of AMD in visually impaired population vs. the General population]. (**B**) Methods of sampling [Probability vs. non-probability sampling]

**Fig. 4:**
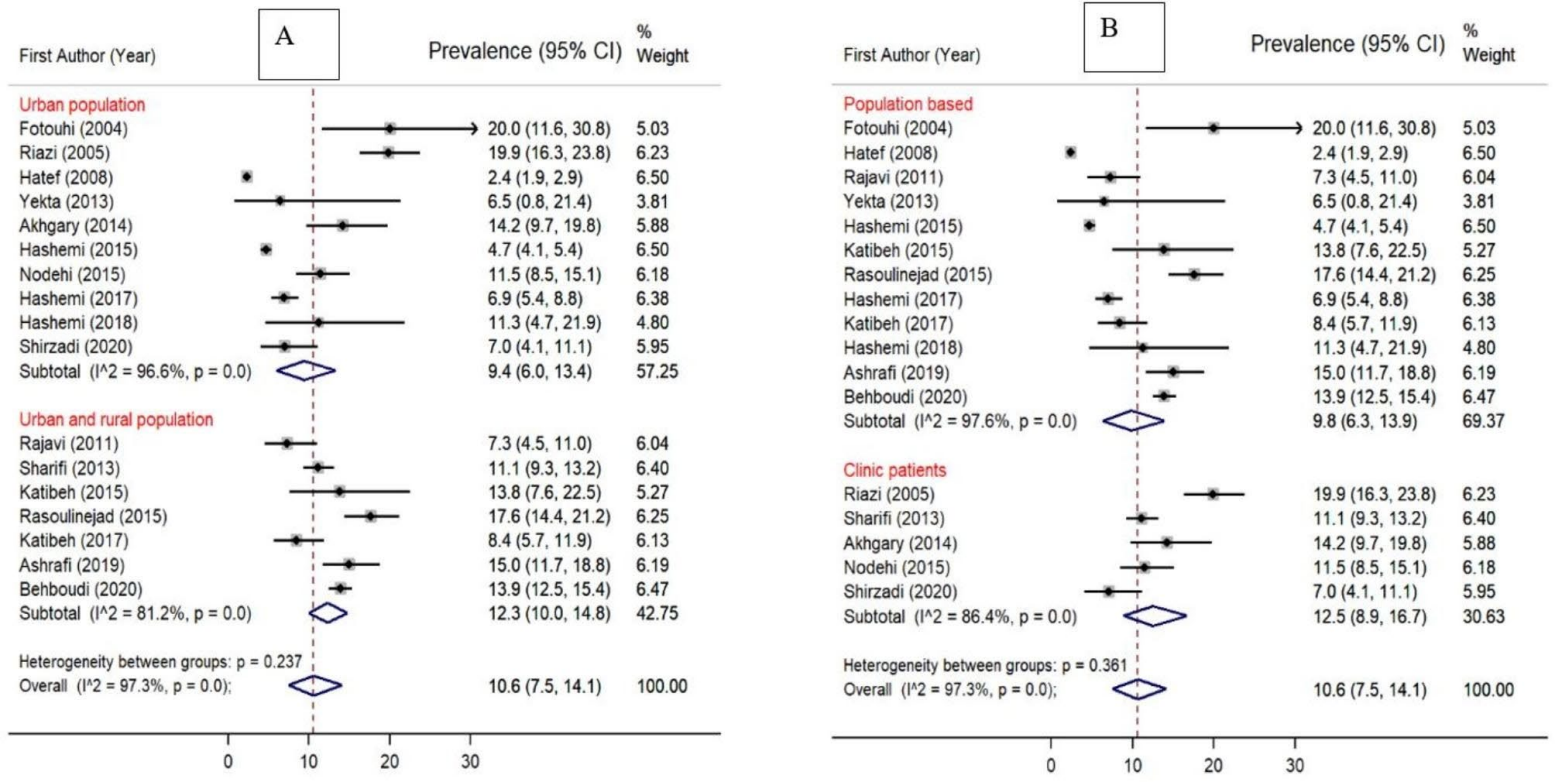
Forest plot diagram showing the subgroup analysis of the prevalence of AMD in the Iranian population and the associated 95% CI in: (**A**) Urban vs. mixed urban and rural population. (**B**) Population-based studies vs. clinic patients

The prevalence of AMD was 11.9% (95% CI [9.3-14.9%]) based on studies among the general or visually impaired population and 8.7% (95% CI [4.5-14.0%]) based on studies among the general population (Fig. 3). Studies with non-probability sampling methods showed a higher prevalence of AMD, compared to studies with probability sampling methods (14.7% vs. 8.9%). Besides, a higher prevalence of AMD was observed in studies conducted in locations with mixed urban and rural populations, compared to studies in only urban populations (12.3% vs. 9.4%) (Fig. 4). Finally, the prevalence of AMD was higher in studies conducted in provinces with medium HDI (12.1%), compared to those with very high (11.3%) and high HDI (9.5%).

**Fig. 5:**
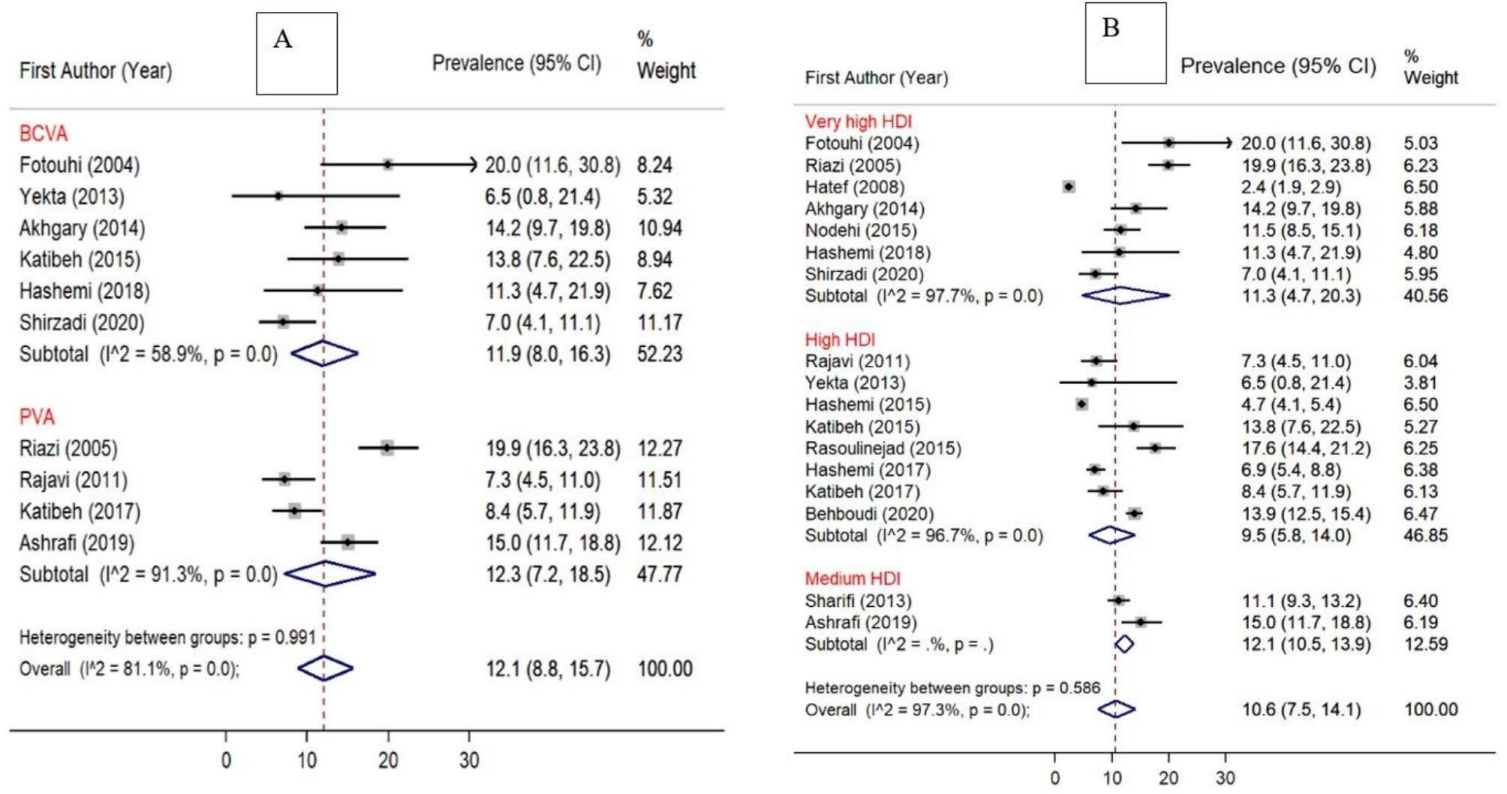
Forest plot diagram showing the subgroup analysis of the prevalence of AMD in the Iranian population and the associated 95% CI based on: (**A**) Assessment methods of the visual acuity. (**B**) HDI of the study location

Forest plots demonstrating subgroup analysis are presented in Figs. 3 and 4, and 5.

We also performed a sensitivity analysis by excluding clinic-based studies and only including population-based studies. The findings of the sensitivity analysis were similar to the primary analysis. The prevalence of AMD was higher in the visually impaired populations (compared to the general populations), in studies conducted in locations with mixed urban and rural populations (compared to studies in only urban populations), and in provinces with medium HDI (compared to those with very high and high HDI). (Figures S1-S4)

### Estimation of the population affected by AMD in Iran from 1990 to 2050

**Fig. 6:**
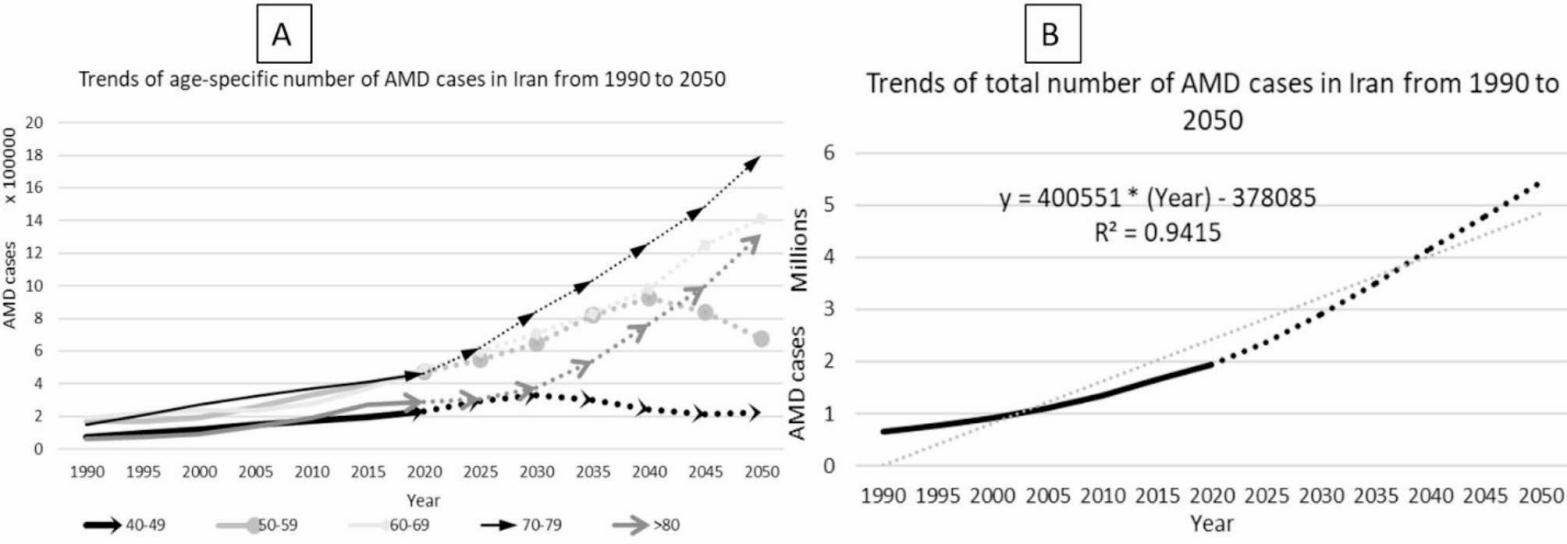
Trends of the estimated number of AMD cases in Iran from 1990 to 2050: (**A**) The age-specific number of AMD cases in Iran. (**B**) The total number of AMD cases in Iran

We observed an increasing trend in the total and age-specific number of cases affected by AMD in Iran from 1990 to 2050. In 1990, 647,420 cases were estimated to be affected by AMD. We observed a three-fold rise in the total number of cases in 2020 compared to 1990, reaching to near two million people. We project that this increasing pattern will continue to 2050, even more rapidly, and the total number of cases will reach to near 5.5 million people. The average annual percent change in total number of AMD cases is 3.66% (95% CI: 3.65–3.67%) from 1990 to 2050. We also demonstrated the age-specific number of cases. It is evident that, by 2050, the population aged 70–79 will constitute the highest proportion of the estimated 5.5 million AMD patients. (Fig. 6).

### Meta-regression of the AMD prevalence

The results of the meta-regression demonstrated that the prevalence of AMD is affected by the method of sampling [coefficient: 1.0535 (1.0015, 1.1082)]. AMD prevalence has also been affected by the location of the study and the mean age of the participants in the population-based studies (Table 3).

**Table 3:** Meta-regression of the prevalence of age-related macular degeneration in the Iranian population based on different variables

Variable	Coefficient (95% CI)		
	All studies	General population^*^	Population-based studies^**^
Publication year	1.0002 (0.9925, 1.0081)	1.0088 (0.9958, 1.0220)	1.0069 (0.9989, 1.0149)
Sampling method	1.0535 (1.0015, 1.1082)^†^	1.0544 (0.9670, 1.1498)	1.0478 (0.9763, 1.1245)
Population based vs. Clinic based	1.0386 (0.9653, 1.1175)	1.0306 (0.8231, 1.2903)	-
General population vs. visually impaired population	1.0403 (0.9742, 1.1107)	-	1.0326 (0.9404, 1.1339)
Mean age	1.0015 (0.9993, 1.0038)	1.0028 (1.0000, 1.0056)^†^	1.0025 (1.0005, 1.0046)^†^
Male to female ratio	1.0001 (0.9989, 1.0013)	1.1698 (0.8911, 1.5355)	1.1427 (0.9284, 1.4065)
HDI	1.0091 (0.9590, 1.0619)	1.0471 (0.9062, 1.2099)	1.0524 (0.9791, 1.1312)
Urban vs. rural	1.0504 (0.9895, 1.1151)	1.1086 (1.0357, 1.1866)^†^	1.0995 (1.0542, 1.1468)^†^

HDI: Human Developmental Index

*The studies in which the target population is not limited to visually impaired participants. In this column, the studies which only evaluate visually impaired population were excluded

**Only population-based studies were evaluated and clinic-based studies were excluded

†Statistically significant (P-value < 0.05)

### Sensitivity analysis and publication bias

Sensitivity analysis was performed by stepwise removal of each study and calculation of pooled prevalence in each step in order to detect the individual effects of any single study. It revealed that the pooled prevalence of AMD did not change significantly after exclusion of each single study in each step (Figure S5). We also evaluated the possibility of publication bias using a funnel plot and Egger’s test. The funnel plot was asymmetric and Egger’s test p-value was 0.028, indicative of the possibility of publication bias (Figure S6).

## Discussion

The overall prevalence of AMD in the general population was near 10% in males and females according to this study. Also, our study showed that the prevalence of AMD is zero in people under the age of 40, but increases linearly by age, reaching one out of three in people over the age of 80. The prevalence of AMD rises with the increase in the severity of vision impairment, from 8.5% in moderate vision impairment to 15.7% in the blind population. We also found that the prevalence of AMD was higher in the studies with non-probability sampling, in areas with medium HDI, and rural populations.

In a meta-analysis by Jonas et al., the prevalence of AMD within an age range of 45–85 was approximately 8.7% globally [36]. In addition, Wong et al. reported a pooled prevalence of AMD of 12.3% in populations of European ancestry and 7.4% in Asians. After correcting for age, the reported prevalence of AMD among European ancestors were 11.6% in the age group 60–69 years, 22.5% in those aged 70–79 years, and 33.6% in those aged 80–84 years. The prevalence of AMD among Asians were 8.3% in the age group 60–69 years, 13.6% in those aged 70–79 years, and 18.9% in those aged 80–84 years [37]. Song et al. found that the prevalence of any AMD in China has a decreasing trend in the last three decades and reached 5.2% in 2015 [38]. The prevalence of AMD in Iranians seems to be somewhere between the prevalence of Asians and Europeans, and the estimated prevalence in different age groups is close to our estimates. The comparison of the results of our meta-analysis with Vanderbeek et al. study [39] revealed that the prevalence of AMD in the age group 50–60 years was 3.5% in the United States and 5.7% in Iran, while the prevalence of AMD among those above 80 years was 40.4% in the United States and 32.3% in Iran. The higher prevalence of AMD in younger age groups might be due to ethnic differences, regional differences, and a discrepancy in the level of social development [40, 41]. Xu et al. found that the burden of AMD, measured by the Disability-adjusted life years (DALY) data gathered from the Global Burden of Disease Study (GBD) 2017, was highest in Eastern Mediterranean and African region and lowest in the Western Pacific region [42]. The higher DALY due to AMD in Eastern Mediterranean region despite a lower prevalence compared to the American region can be attributed to the higher prevalence of AMD in younger age groups in the Eastern Mediterranean region, as described above [37, 39]. In addition, it is found that lower socioeconomic status and education levels are associated with higher disease burden [42]. People with higher levels of education are more likely to pursue ophthalmic treatment and higher socioeconomic regions have superior resources to offer high-quality ophthalmic care [42].

The results from our study showed that the prevalence of AMD was not different between males and females. Although in a previous meta-analysis, the female sex was considered a weak risk factor for late AMD [43], based on Jonas et al. and Wong et al.‘s studies [36, 37], gender was not associated with the prevalence of AMD or with the frequency of AMD as a cause for vision impairment or blindness which is consistent with our results.

This meta-analysis showed that the prevalence of AMD was higher in regions with medium HDI, compared to provinces with high and very high HDI. Besides, a higher prevalence of AMD was observed in studies conducted in locations with mixed urban and rural populations, compared to studies in only urban populations (12.3% vs. 9.4%). Previous reports from India and Italy also found that the prevalence of AMD was higher in rural areas and regions with medium socioeconomic status [44, 45]. Also, a report by Xu et al. demonstrated that the burden of macular degeneration was correlated inversely with socioeconomic status, HDI, and education level [42]. More difficult access to health care systems and lower prevalence of insurance coverage and health-related awareness in rural areas has yielded to the rural-urban disparity in the prevalence of many diseases, including diabetes and coronary heart diseases, which can be similarly applied to AMD [46, 47]. However, there is still some controversy about the impact of living in rural areas and the prevalence of AMD. For instance, in a recent meta-analysis of the Chinese population, AMD was found to be more prevalent in urban populations than in rural populations [48].

The results of the subgroup analysis revealed that there is a slight and ignorable difference between the prevalence of AMD based on BCVA (11.9%) and PVA (12.3%). Although the difference is not significant (p-value = 0.79), further investigations should evaluate both BCVA and PVA when reporting AMD-related visual impairment [1]. Further subgroup analysis based on the vision status of the participants revealed that the prevalence of AMD is higher among the patients with more severe vision impairment. The prevalence of AMD was 8.5% in moderate vision impaired, 13.6% in severe vision impaired population, and 15.7% in blind participants. These results are consistent with the findings of a previous study by Nangia et al., demonstrating that the proportion of people affected by AMD was 9.6% in the moderate or severe vision impaired and 16.4% in the blind population in 2020 [49]. In another study, AMD caused 5.6% of the total age-standardized prevalence of blindness in 2020 and it was the greatest contributor in the oldest age group [3]. Furthermore, AMD accounts for 3.0% of the age-standardized prevalence of moderate and severe vision impairment, which makes AMD the third most common cause of vision impairment, after refractive errors and cataracts [3].

These results indicate a rise in the relative frequency of AMD as the severity of vision impairment increases in a population. This finding can be attributed to the degenerative and progressive nature of AMD, which can cause severe vision impairment and blindness if left untreated [1].

In the current meta-analysis, studies with non-probability sampling methods showed a higher prevalence of AMD compared to studies with probability sampling methods (14.7% vs. 8.9%). Moreover, the prevalence of AMD in population-based studies was 9.8% versus 12.5% in studies based on clinic patients. Non-probability sampling methods are more convenient ways to collect data; however, they might endanger the representativeness and generalizability of the sample [13]. In our meta-analysis, the studies with non-probability sampling were mostly in clinic settings, while the studies with probability sampling methods were population-based. It is predictable that people attending the ophthalmology clinics are more prone to be affected by AMD, than the normal population. Therefore, sampling methods should be considered by future studies before generalizing the findings to the whole population.

To the best of our knowledge, this study is the first to estimate the prevalence of AMD in Iran and make future projections. Our comprehensive search strategy helped us to identify and include studies from different age groups; therefore, we were able to report the prevalence of AMD among different age groups and estimate the age-specific and total number of AMD cases. By the year 2050, we estimate that an estimated 5.5 million people will be affected by AMD, and patients aged 70–79 will constitute the highest proportion. Similar increasing trends in the estimated number of AMD cases were observed in a study from China. Song et al. found that the total number of AMD cases in China rises significantly from 12.0 million in 1990 to 26.6 million in 2015 and to 55.2 million in 2050 [38]. As a result, it is vital to address the importance of primary prevention, such as nutritional supplements such as the Age-Related Eye Disease Study (AREDS) formula, lifestyle modifications like smoking cessation, and using sunglasses [50]. The information provided is essential for shaping clinical and public health policies and offers valuable perspectives on the impact of AMD in Iran. As a result, it can serve as a foundation for health policy development and the allocation of resources toward the prevention and treatment of AMD.

Our study has several strengths and advantages. First, to the best of our knowledge, this meta-analysis is the first one conducted on the Iranian population with a large sample size and a comprehensive search strategy. Second, subgroup analyses were done based on visual impairment, sampling methods, HDI, and geographical locations. There are limitations of this study that should be noted before drawing any conclusions. Firstly, significant heterogeneity existed between all of the included studies, despite our strict inclusion and exclusion criteria. However, heterogeneity in prevalence estimates is highly expected when conducting a country-wide systematic review of prevalence, with many studies varying in the study year, location, populations, and methodologies. Like any meta-analysis, the quality of the primary studies might have affected the robustness of our methodology to some point. Secondly, not all the included studies report the gender-specific prevalence and whole population prevalence of AMD. Furthermore, most of the studies did not report their result based on early and late AMD.

## Conclusion

In summary, this systematic review and meta-analysis provides a comprehensive and up-to-date estimated prevalence of AMD among Iranians. The prevalence of AMD was near 10% in general population, and this seems to be somewhere between the prevalence of Asians and Europeans. Considering the aging population in Iran and a nearly three-fold increase in the total number of cases by 2050, it is crucial to address the importance of primary prevention and the use of this information for future policymaking.

### Electronic supplementary material

Below is the link to the electronic supplementary material.

Supplementary Material 1

## Data Availability

The datasets used and/or analyzed during the current study are available from the corresponding author on reasonable request.
